# 2495 Respiratory therapists’ awareness and intention to use the electronic modified early warning score (eMEWS)

**DOI:** 10.1017/cts.2018.224

**Published:** 2018-11-21

**Authors:** Constance Mussa, Afnan Al-Raimi

## Abstract

OBJECTIVES/SPECIFIC AIMS: To determine if an educational intervention designed to increase respiratory therapists’ knowledge of the modified early warning score (MEWS) would influence their intention to use the MEWS. METHODS/STUDY POPULATION: A web-based self-administered survey based on the constructs of the TAM as well as awareness, attitude, and job-relevance was developed and validated using traditional scale development process and distributed to 75 respiratory therapists (RTs) from the respiratory care department of Rush University Medical Center. RTs were recruited for participation in the study using consecutive sampling. The RTs were then given a training session on the MEWS after which they were again asked to complete the survey. RESULTS/ANTICIPATED RESULTS: The response rate to both the pre and post survey was 60 percent. Of the 46 participants recruited to the study, the educational intervention elicited an increase in the MEWS knowledge score in 45 participants compared with the knowledge score prior to the educational intervention. Additionally, there was an increase in the behavioral intention score post intervention in 30 participants compared with the behavioral intention score before the educational intervention. A Wilcoxon signed-rank test determined that there was a statistically significant median increase in MEWS knowledge score (2.0) post educational intervention (4.0) compared with pre-educational intervention (2.0), *p*<0.0005. There was also a statistically significant median increase in behavioral intention score (0.667) pre-educational intervention (4.0) compared with posteducational intervention (3.0), *p*<0.0005. DISCUSSION/SIGNIFICANCE OF IMPACT: Numerous studies over the last 4 decades have demonstrated that change in behavioral intention is a good predictor of change in behavior. Consequently, the increase in the respiratory therapists’ behavioral intention score post MEWS education suggests that they may be more inclined to incorporate the MEWS score in their assessment of patients if they are educated about its clinical relevance. Additionally, the study results verified key postulates of the TAM, suggesting that the TAM is an appropriate model for assessing respiratory therapists’ perception and reaction to new systems, and may also help respiratory care managers develop new mechanisms that facilitate respiratory therapists’ adoption of new systems and processes.

